# The Bet v 1 fold: an ancient, versatile scaffold for binding of large, hydrophobic ligands

**DOI:** 10.1186/1471-2148-8-286

**Published:** 2008-10-15

**Authors:** Christian Radauer, Peter Lackner, Heimo Breiteneder

**Affiliations:** 1 Department of Pathophysiology, Medical University of Vienna, Währinger Gürtel 18-20, 1090 Vienna, Austria; 2 Department of Molecular Biology, University of Salzburg, Hellbrunnerstraße 34, 5020 Salzburg, Austria

## Abstract

**Background:**

The major birch pollen allergen, Bet v 1, is a member of the ubiquitous PR-10 family of plant pathogenesis-related proteins. In recent years, a number of diverse plant proteins with low sequence similarity to Bet v 1 was identified. In addition, determination of the Bet v 1 structure revealed the existence of a large superfamily of structurally related proteins. In this study, we aimed to identify and classify all Bet v 1-related structures from the Protein Data Bank and all Bet v 1-related sequences from the Uniprot database.

**Results:**

Structural comparisons of representative members of already known protein families structurally related to Bet v 1 with all entries of the Protein Data Bank yielded 47 structures with non-identical sequences. They were classified into eleven families, five of which were newly identified and not included in the Structural Classification of Proteins database release 1.71. The taxonomic distribution of these families extracted from the Pfam protein family database showed that members of the polyketide cyclase family and the activator of Hsp90 ATPase homologue 1 family were distributed among all three superkingdoms, while members of some bacterial families were confined to a small number of species. Comparison of ligand binding activities of Bet v 1-like superfamily members revealed that their functions were related to binding and metabolism of large, hydrophobic compounds such as lipids, hormones, and antibiotics. Phylogenetic relationships within the Bet v 1 family, defined as the group of proteins with significant sequence similarity to Bet v 1, were determined by aligning 264 Bet v 1-related sequences. A distance-based phylogenetic tree yielded a classification into 11 subfamilies, nine exclusively containing plant sequences and two subfamilies of bacterial proteins. Plant sequences included the pathogenesis-related proteins 10, the major latex proteins/ripening-related proteins subfamily, and polyketide cyclase-like sequences.

**Conclusion:**

The ubiquitous distribution of Bet v 1-related proteins among all superkingdoms suggests that a Bet v 1-like protein was already present in the last universal common ancestor. During evolution, this protein diversified into numerous families with low sequence similarity but with a common fold that succeeded as a versatile scaffold for binding of bulky ligands.

## Background

Plants are continuously challenged by pathogens, herbivores and adverse environmental conditions. Although they lack an adaptive immune system, they have evolved numerous other mechanisms of stress response and defence. These include developmental plasticity, mechanical barriers, low molecular weight anti-microbial compounds named phytoalexins, pathogen-specific resistance genes and inducible pathogenesis-related (PR) proteins. PR-proteins were first discovered as proteins present in tobacco plants infected with tobacco mosaic virus [1]. They were defined as proteins absent in the non-infected plant and induced after pathogen infection or other adverse conditions. PR-proteins are today classified into 17 families based on sequence similarity and biochemical function [2].

An unusual PR family was designated PR-10 or intracellular PR-proteins as they are, in contrast to most PR families, expressed in the cytoplasm. They are acidic proteins of 15–18 kDa and were first discovered in parsley and peas in the late 1980s [3,4]. Since then, PR-10 family members have been found in many species of higher plants ranging from conifers to monocotyledonous and dicotyledonous angiosperms [5]. In 1989, this protein family became a target of research in a completely different area when the major pollen allergen of white birch (*Betula verrucosa*), Bet v 1, was cloned and its sequence revealed to be similar to PR-10 proteins (55% identity and 70% similarity to a pea disease resistance protein) [6]. In subsequent years, homologous allergens from pollen of related trees such as alder and hazel as well as food allergens from fruits and vegetables such as apple and celery were identified. Immunoglobulin E cross-reactivity among these allergens is responsible for the frequent occurrence of plant food allergy among birch pollen allergic individuals, an association termed the birch-fruit syndrome [7].

In 1998, the finding that a group of proteins abundantly expressed in the latex of opium poppy was homologous to PR-10 proteins despite sequence identities below 25% revealed that the PR-10 family was part of a much larger family of plant proteins [8]. Since then, several other proteins distantly related to PR-10 proteins were described. (S)-Norcoclaurine synthases (NCS), enzymes involved in benzylisoquinoline alkaloid biosynthesis, from poppy [9] and meadow rue (*Thalictrum flavum*) [10] showed sequence identities to PR-10 proteins between 28% and 38% [10]. The sequence of a cytokinin-specific binding protein (CSBP) from mung bean displayed 31% identity and 45% similarity to Bet v 1 [11].

The most striking feature of the three-dimensional structure of Bet v 1 is the presence of a large hydrophobic cavity, which is open to the exterior and probably functions as a ligand binding site [12]. The surprising similarity of the structure of the Bet v 1-related major cherry allergen, Pru av 1, to that of the lipid binding domain of the human cholesterol-binding metastatic lymph node protein 64 (MLN64) revealed the existence of a large superfamily of mostly lipid-binding proteins with a common fold [13]. This superfamily was classified as Bet v 1-like clan in the Pfam protein family database [PfamC:CL0209] and as Bet v 1-like superfamily in the Structural Classification of Proteins (SCOP) database [SCOP:d.129.3].

In this study, we aimed, on the one hand, to classify all available sequences with significant similarity to Bet v 1 into subfamilies and, on the other hand, to determine all structural Bet v 1 homologues in the Protein Data Bank (PDB) using a sensitive structural alignment tool. These structures were classified into eleven families adding five families to the six families included in SCOP release 1.71. The ubiquitous taxonomic distribution of Bet v 1-like superfamily members and their ligand binding activities suggest that the Bet v 1 fold can be traced back to the last universal common ancestor and that the primordial Bet v 1-like protein probably functioned as a lipid carrier.

## Results and discussion

### Protein families structurally related to Bet v 1

In order to expand the knowledge of Bet v 1-related structures beyond those already classified in SCOP, we performed structural alignments of representative members of all Bet v 1-related families to all PDB entries. This search yielded 47 structures with non-identical sequences including isoforms and engineered mutants that were classified into 11 families (Tables 1 and 2, Additional file 1). The structures were classified by structural similarity (defined as the number of aligned residues) and sequence similarity by performing sequence similarity searched against the Uniprot and Pfam databases. In addition to the six SCOP families included in SCOP release 1.71, five new families that contained only one or two structures were defined as members of the Bet v 1-like superfamily.

**Table 1:** Structural classification of the Bet v 1-like superfamily.

Family name	SCOP family	No. of structures^a^	Representative structure		
			PDB ID	Description	RMSD/no. of aligned residues^b^
Bet v 1 family	Pathogenesis-related protein 10 (PR10)-like [SCOP:d.129.3.1]	15	[PDB:1bv1]	Birch pollen allergen Bet v 1; *Betula verrucosa *(white birch)	0.00/159
START domain	STAR domain [SCOP:d.129.3.2]	3	[PDB:1em2]	STAR-related lipid transport domain of MLN64; *Homo sapiens *(human)	2.43/108
Ring hydroxylases α-chain	Ring hydroxylating alpha subunit catalytic domain [SCOP:d.129.3.3]	9	[PDB:1o7n]	Naphthalene 1,2-dioxygenase; *Pseudomonas putida*	2.42/86
Phosphatidylinositol transfer proteins	Phosphatidylinositol transfer protein, PITP [SCOP:d.129.3.4]	4	[PDB:1t27]	Phosphatidylinositol transfer protein alpha; *Rattus norvegicus *(rat)	2.28/111
AHA1 domain	Aha1 domain [SCOP:d.129.3.5]	8	[PDB:1xfs]	Hypothetical protein NE0264; *Nitrosomonas europaea*	2.31/115
Polyketide cyclases	Oligoketide cyclase/dehydrase-like [SCOP:d.129.3.6]	2	[PDB:1t17]	Hypothetical protein CC1736; *Caulobacter crescentus*	2.59/114
SMU440-related	-	1	[PDB:2b79]	Hypothetical protein SMU440; *Streptococcus mutans*	2.65/132
PA1206-related	-	1	[PDB:2ffs]	Hypothetical protein PA1206; *Pseudomonas aeruginosa*	2.53/115
Homotrimeric ring hydroxylases	-	2	[PDB:1z01]	2-Oxo-1,2-dihydroquinoline 8-monooxygenase; *Pseudomonas putida*	2.47/99
CalC-related	-	1	[PDB:1zxf]	Self-sacrificing resistance protein CalC; *Micromonospora echinospora*	2.93/87
CoxG family	-	2	[PDB:2ns9]	Hypothetical protein APE2225; *Aeropyrum pernix*	2.53/117

^a^Number of structures of proteins with non-identical sequences in PDB.

^b^Root mean square distance of backbone atoms of all aligned residues and number of aligned residues in a structural alignment with Bet v 1 [PDB:1bv1].

**Table 2:** Pfam families corresponding to structural Bet v 1-related families.

Family name	Pfam family	Number of sequences in Pfam	Range of percent sequence identity/similarity to Bet v 1
Bet v 1 family	Pathogenesis-related protein Bet v I family [Pfam:PF00407]	520	9–100/15–100
START domain	START domain [Pfam:PF01852]	522	1–20/1–30
Ring hydroxylases α-chain	Ring hydroxylating alpha subunit (catalytic domain) [Pfam:PF00848]	1156	0–19/0–33
Phosphatidylinositol transfer proteins	Phosphatidylinositol transfer protein [Pfam:PF02121]	104	1–16/1–27
AHA1 domain	Activator of Hsp90 ATPase homolog 1-like protein [Pfam:PF08327]	617	0–23/0–35
Polyketide cyclases	Polyketide cyclase/dehydrase and lipid transport [Pfam:PF03364]	908	0–22/1–35
SMU440-related	[PfamB:PB094079]	2	4–19/9–33
PA1206-related	Domain of unknown function (DUF1857) [Pfam:PF08982]	36	3–22/4–38
Homotrimeric ring hydroxylases	[PfamB:PB024837]	7	10–14/19–24
CalC-related	[PfamB:PB077055] + [PfamB:PB048144]	3	8–17/12–32
CoxG family	Carbon monoxide dehydrogenase subunit G (CoxG) [Pfam:PF06240]	176	1–24/2–38

#### Bet v 1 family

**Figure 1:**
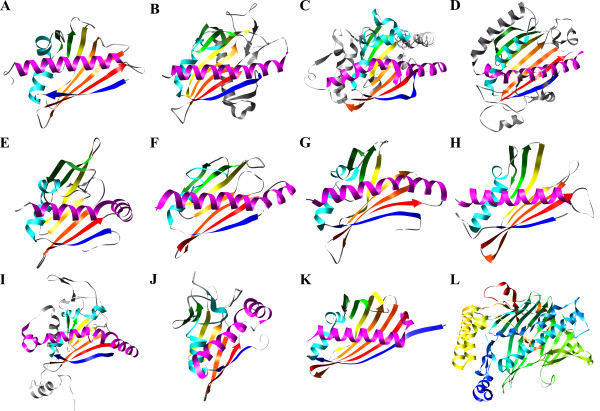
**Structures of representative members of the Bet v 1-like superfamily (A-K) and of oxysterol binding protein (L)**. **A: **Birch pollen allergen Bet v 1, *Betula verrucosa *[PDB:1bv1]; **B: **STAR-related lipid transport domain of MLN64, *Homo sapiens *[PDB:1em2]; **C: **Naphthalene 1,2-dioxygenase, α-chain, C-terminal domain, *Pseudomonas putida *[PDB:1o7n]; **D: **Phosphatidylinositol transfer protein alpha, *Rattus norvegicus *[PDB:1t27]; **E: **Hypothetical protein NE0264, *Nitrosomonas europaea *[PDB:1xfs], a member of the AHA1 family; **F: **Hypothetical protein CC1736, *Caulobacter crescentus *[PDB:1t17], a member of the polyketide cyclase family; **G: **Hypothetical protein SMU.440, *Streptococcus mutans *[PDB:2b79]; **H: **Hypothetical protein PA1206, *Pseudomonas aeruginosa *[PDB:2ffs]; **I: **2-Oxo-1,2-dihydroquinoline 8-monooxygenase, C-terminal domain, *Pseudomonas putida *[PDB:1z01], a homotrimeric ring hydroxylase; **J: **Self-sacrificing resistance protein CalC, *Micromonospora echinospora *[PDB:1zxf]; **K: **Hypothetical protein APE2225, *Aeropyrum pernix *[PDB:2ns9], a member of the CoxG family. **L: **Oxysterol-binding protein, *Saccharomyces cerevisiae *[PDB:1zht]. Structures in A-K were coloured according to secondary structure elements homologous to Bet v 1. L was coloured by chain position from blue (N-terminus) to red (C-terminus). Images were generated with UCSF Chimera [61].

The family containing the highest number of structures was the Bet v 1 family [SCOP:d.127.3.1] with 15 structures with non-identical sequences. Members of this family fold into the prototypic Bet v 1 structure with the secondary structure arrangement β-α_2_-β_6_-α with an anti-parallel β-sheet (topology 1765432) wrapped around a long C-terminal α-helix (Fig. 1A). Subfamilies of the Bet v 1 family [Pfam:PF00407] are discussed in detail below. The PDB contained 12 structures of PR-10 subfamily members and one structure each of a major latex protein (MLP) and a CSBP (Additional file 1). In addition, the structure of an uncharacterized protein from *Arabidopsis thaliana *[PDB:1vjh] with distant sequence similarity to MLPs (36% identity, 50% similarity to a Bet v 1 family member from *Brassica campestris *[Uniprot:A8IXG5]) can be classified as Bet v 1-related, although it has a large sequence deletion resulting in the lack of two β-strands.

#### START family

Three structures were classified into the START family [SCOP:d.127.3.2], named after the steroidogenic acute regulatory (STAR) protein-related lipid transfer (START) domain: the START domain of the human STAR-related lipid transfer protein 3, also termed MLN64, mouse STAR-related lipid transfer protein 4, and human phosphatidylcholine transfer protein (Additional file 1). The structure of the START domain differs from the Bet v 1 structure by an N-terminal extension consisting of an α-helix on the outer face of the β-sheet and two additional β-strands adding to the β-sheet on the side of the N-terminal strand (Fig. 1B). START domains occur in a wide range of diverse, lipid-binding proteins. Pfam contained 522 sequences from bacteria and eukaryotes matching the START family signature [Pfam:PF01852]. The prototypic member of this family, STAR, stimulates the transport of cholesterol from the outer to the inner mitochondrial membrane, where it is required as a substrate for steroidogenic enzymes [14]. Other family members include ceramide transfer proteins [15] and phosphatidylcholine transfer proteins [16]. Multi-domain proteins that contain a START domain are mammalian cytosolic acetyl-CoA hydrolases [17] and some members of the HD-ZIP family of homeodomain transcription factors from plants [18].

#### Ring hydroxylases α-chain

The PDB contained structures of 9 different wild-type or mutant enzymes from the aromatic ring hydroxylase family ([SCOP:d.129.3.3]; Additional file 1). This family of bacterial enzymes catalyses the first step in the degradation of polycyclic aromatic compounds by converting their substrates into non-aromatic *cis*-dioles [19]. These multi-component protein complexes consist of a ferredoxin reductase, a ferredoxin, and a hexameric hydroxylase with the subunit composition α_3_β_3_. The catalytic α-subunit contains an N-terminal Rieske domain with a 2Fe-2S-cluster and a C-terminal Bet v 1-like domain that binds a ferrous ion. Their Bet v 1-like domains contain a β-sheet extended to 9 strands and several additional helices inserted at the N and C-termini and at an internal site (Fig. 1C). The hydroxylases are the largest family within the Bet v 1-like superfamily with 1156 sequences recorded in Pfam [Pfam:PF00848].

#### Phosphatidylinositol transfer proteins

Four structures were classified into the phosphatidylinositol transfer protein (PITP) family ([SCOP:d.129.3.4], Additional file 1). These eukaryotic proteins transfer phosphatidylinositol and phosphatidylcholine between membranes. Their structures differ from that of Bet v 1 by an internal extension containing several short helices and a β-strand parallel to the first strand of the Bet v 1-like sheet and a C-terminal extension that comprises a second long helix and a short stretch that closes the ligand-binding cavity in the ligand-bound conformation (Fig. 1D). The hydrophobic ligand-binding pocket is formed by the Bet v 1-like core. The α-helical region, known as the regulatory loop, contains a protein kinase C phosphorylation site. The C-terminus can undergo large conformational changes between a closed, ligand-bound structure, and an open, membrane bound structure that enables phospholipid binding and release [20]. Pfam contained 104 PITP sequences [Pfam:PF02121], most of them from animals. Mammalian PITPs were classified by sequence similarity into five subfamilies, three (PITPα, PITPβ, RdgBβ) containing soluble single-domain proteins and two (RdgBαI, RdgBαII) containing large multi-domain proteins with a PITP domain at the N-terminus [20].

#### AHA1 family

The activator of Hsp90 ATPase homolog 1 (AHA1, p38) interacts with the middle domain of heat shock protein 90 and stimulates its ATPase activity [21]. Its C-terminal domain is structurally related to Bet v 1. The PDB contained structures of eight members of the AHA1 family [SCOP:d.129.3.5], the C-terminal domain of human AHA1 as well as one archaeal and six bacterial proteins of unknown function (Additional file 1). The fold of the AHA1-like domain is identical to the Bet v 1 fold except an additional N-terminal strand present in some family members, which pairs with the strand homologous to strand 1 in Bet v 1 (Fig. 1E). Proteins from the AHA1 Pfam family [Pfam:PF08327] were found in eukaryotes, bacteria, and archaea with 617 sequences recorded in Pfam. While the domain organisation of most eukaryotic homologues matches that of AHA1 suggesting a similar function, a wide range of diverse prokaryotic proteins with AHA1-related domains were found in Pfam. Among those were members of the ArsR family, a group of metallosensitive transcription repressors [22].

#### Polyketide cyclases

The polyketide cyclase family [SCOP:d.129.3.6] was named after family members with polyketide cyclase and dehydrase activity found in *Streptomyces *species [23]. The PDB contained two structures of bacterial polyketide cyclase-like predicted proteins from *Caulobacter crescentus *and *Thermus thermophilus *(Additional file 1). Their folds are identical to the Bet v 1 fold (Fig. 1F) despite low sequence identities to Bet v 1 in a global alignment (16% and 20% identity, 30% and 35% similarity). Polyketide cyclases catalyse the cyclisation of polyketides (poly-β-keto adducts of short-chain carboxylic acids) in the biosynthesis of a diverse group of compounds including pigments, antibiotics and anti-tumour drugs [24]. There are several unrelated families of polyketide cyclases of which one is a member of the Bet v 1-like superfamily [Pfam:PF03364]. Proteins from this family are ubiquitously found in bacteria, archaea and eukaryotes forming the second largest family within the Bet v 1-like superfamily with 908 sequences from 449 species, most of them proteins of unknown function, annotated in the Pfam database. The majority of the members of this family most likely possess functions other than polyketide synthesis as exemplified by the CoQ10 family of coenzyme Q binding proteins from yeast and mammals (e. g. [Uniprot:Q08058], [Uniprot:Q96MF6]).

#### SMU440-related proteins

The structure of the predicted protein of unknown function SMU440 from *Streptococcus mutans *[PDB:2b79] closely matches that of Bet v 1 (Fig. 1G), but shows only low sequence similarity (19% identity, 33% similarity in a global alignment). It shares 23–46% sequence identity (38–69% similarity) with a small family of uncharacterized bacterial proteins and is classified in Pfam into a non-annotated PfamB family [PfamB:PB094079].

#### PA1206-related proteins

Another uncharacterized bacterial protein with a fold identical to Bet v 1 is PA1206 from *Pseudomonas aeruginosa *([PDB:2ffs], Fig. 1H). It is a member of a Pfam family [Pfam:PF08982] of proteins with unknown functions that contained 36 sequences from bacteria and fungi.

#### Homotrimeric ring hydroxylases

A group of aromatic hydrocarbon hydroxylating enzymes that includes 2-oxo-1,2-dihydroquinoline 8-monooxygenase from *Pseudomonas putida *and carbazole 1,9a-dioxygenase from *Janthinobacterium *was classified into its own family (Additional file 1). These enzymes are homotrimers with subunits similar to the α-subunits of typical ring hydroxylating oxygenases composed of an N-terminal Rieske domain that binds an iron-sulphur cluster, and a C-terminal Bet v 1-like domain [25]. In contrast to the typical oxygenases, they lack a β-subunit. Sequences of their C-terminal domains are only distantly related to typical oxygenases (0–17% identity, 0–31% similarity in a global alignment of partial sequences that match the Pfam family signatures). They do not match the Ring_hydroxyl_A family signature [Pfam:PF00848], but a non-annotated PfamB domain [PfamB:PB024837]. Despite similar enzymatic functions, structures of members of these families show considerable differences (compare Figs. 1C and 1I). While the position of insertions to the Bet v 1-like core is identical for both families (between β-strands 1 and 2, between strands 3 and 4, and at the C-terminus), the lengths and secondary structure contents of the insertions differ. The C-terminal extension of regular hydroxylases contains two β-strands that extend the sheet to a nine-stranded one. In contrast, homotrimeric hydroxylases contain two additional two-stranded sheets formed by the first and second insertion.

#### CalC-related proteins

The protein CalC from *Micromonospora echinospora *confers resistance to the enediyne antibiotic calicheamicin [26]. Enediynes are cytotoxic compounds whose binding to DNA results in activation and oxidative cleavage of the DNA strands. CalC was shown to bind calicheamicin with high affinity thereby being cleaved at a specific glycine residue, a mechanism termed self-sacrifice resistance [26]. A BLAST search yielded 86% identity (90% similarity) to a predicted protein from the closely-related actinomycete *Salinispora arenicola *and 25–36% identity (43–55% similarity) to uncharacterised bacterial members of the AHA1 family. The fold of CalC differs from the Bet v 1 fold by an unstructured N-terminus of 30 residues and the disruption of the C-terminal helix by a non-helical region of five residues in length (Fig. 1J).

#### CoxG family

The genomes of aerobic autotrophic bacteria that utilise carbon monoxide as a carbon source contain the *Cox *gene cluster that contains the genes encoding the subunits of the carbon monoxide dehydrogenase and several accessory genes. One of these, *CoxG*, is a member of the Bet v 1-like superfamily. The PDB contained two structures classified into the CoxG family: predicted proteins from the bacterium *Geobacillus kaustophilus *and the archaeon *Aeropyrum pernix *(Additional file 1; Fig. 1K). Their folds are identical to the Bet v 1 fold despite the lack of significant sequence similarity (13% and 14% identity, 25% similarity in a global alignment). The function of the CoxG gene product was determined by gene disruption in *Oligotropha carboxidovorans *as anchoring the CO dehydrogenase to the cytoplasmic membrane [27]. The Pfam database [Pfam:PF06240] contained 176 sequences from bacteria and archaea, most of them predicted proteins of unknown function.

### Sequence similarities between members of different Bet v 1-related families

Members of the Bet v 1-like superfamily from families other than the Bet v 1-family showed sequence identities to Bet v 1 below 25% and similarities below 40% in a global alignment (Table 2). An all-against-all comparison of representative sequences from all Bet v 1-related families yielded median sequence identities below 15% and median similarities below 20% for most pairs of families (data not shown). The families whose members showed the highest degree of sequence similarity were the AHA1 and CalC families (18% median identity, 29% median similarity) and the Polyketide cylcase and CoxG families (16% median identity, 28% median similarity).

### Oxysterol binding proteins – a case of convergent evolution

The structural similarity search among all PDB entries yielded three structures of an oxysterol-binding protein from yeast ([PDB:1zht], [PDB:1zhy], [PDB:1zhz], Fig. 1L). Similarly to Bet v 1-like superfamily members, this protein contains a binding pocket for steroid ligands formed by a long α-helix and an anti-parallel β-sheet. However, the topology of this structure is different with its α-helix at the N-terminus and the β-strands connected in sequential order. The existence of this fold suggests that the structural motif of an anti-parallel β-sheet wrapped around a long α-helix is a structurally stable solution of the evolutionary challenge of developing a large, hydrophobic pocket for lipid binding.

### Taxonomic distribution of Bet v 1-related families

Table 3 summarises the taxonomic distribution of Pfam families determined to be members of the Bet v 1-like superfamily. The two most widely distributed families are the polyketide cyclase family with 908 sequences from 449 species and the AHA1 family with 617 sequences from 252 species. These families are the only ones distributed in all three superkingdoms. Other widely distributed families are the ring hydroxylases and the CoxG family in bacteria and archaea and the START family in bacteria and eukaryotes. Two large families are confined to eukaryotes, the Bet v 1 family, whose members are found exclusively in plants, and the phosphatidylinositol transfer proteins. Several bacterial families are distributed only among a small number of species which suggests that these proteins developed from a primordial Bet v 1-like gene by adopting a specialised function as exemplified by the antibiotic resistance protein CalC.

**Table 3:** Taxonomic distribution of Bet v 1-related Pfam families.

Taxon	Bet v I [Pfam: PF00407]	START [Pfam: PF01852]	Ring hydroxyl. α-chain [Pfam: PF00848]	PITP [Pfam: PF02121]	AHA1 [Pfam: PF08327]	Polyketide cyclase [Pfam: PF03364]	SMU440 [PfamB: PB094079]	PA1206 [Pfam: PF08982]	Homotrimeric ring hydroxyl. [PfamB: PB024837]	CalC [PfamB: PB077055]	CoxG [Pfam: PF06240]
Eukaryota											
Alveolata		✓		✓	✓	✓					
Diplomonadida		✓		✓							
Entanoebidae		✓			✓						
Euglenozoa		✓			✓	✓					
Fungi		✓		✓	✓	✓		✓			
Metazoa		✓		✓	✓	✓					
Mycetozoa				✓	✓	✓					
Parabasalidea		✓		✓							
Viridiplantae	✓	✓		✓	✓	✓					

Bacteria											
Acidobacteria					✓	✓					✓
Actinobacteria			✓		✓	✓					✓
Bacteroidetes		✓			✓	✓					
Chlorobi						✓					
Chloroflexi		✓			✓	✓					✓
Cyanobacteria					✓	✓					
Deinococcus-Thermus					✓	✓					✓
Firmicutes			✓		✓	✓	✓				✓
Fusobacteria							✓			✓	
Planctomycetes						✓					
Proteobacteria		✓	✓		✓	✓		✓	✓		✓
Spirochaetes					✓						
Thermotogae					✓						

Archaea											
Crenarchaeota			✓			✓					✓
Euryarchaeota					✓	✓					✓

### Ligand binding activities

The most distinctive feature of the Bet v 1 fold is a large solvent accessible hydrophobic cavity, which may function as a ligand binding site. In Table 4, experimentally determined ligands of Bet v 1-like superfamily members are shown. Most ligands can be classified as bulky and hydrophobic.

**Table 4:** Experimentally determined ligands and substrates of members of the Bet v 1-like superfamily.

Ligand	Family	Subfamily, Protein	References
Membrane lipids			
phosphatidylcholine	START	phosphatidylcholine transfer protein	[16]
	PITP	PITP	[20]
phosphatidylinositol	PITP	PITP	[20]
ceramide	START	ceramide binding proteins	[15]
cholesterol	START	STAR	[14]
Plant hormones			
brassinosteroids	Bet v 1	PR-10	[13,35]
cytokinins	Bet v 1	CSBP (*Vigna radiata*)	[11]
		Bet v 1, PR-10c (*Betula verrucosa*)	[36,37]
		UBP34 (*Physcomitrella patens*)	[38]
Secondary metabolites			
flavonoids	Bet v 1	Bet v 1, PR-10c (*Betula verrucosa*)	[36,37]
dopamine + 4-hydroxyphenylacetaldehyde	Bet v 1	(S)-norcoclaurine synthases	[9]
emodin	Bet v 1	Hyp-1 (*Hypericum perforatum*)	[39]
polyketides	Polyketide cyclases	Polyketide cyclases (*Streptomyces spp.*)	[23]
enediynes	CalC-related	CalC (*Micromonospora echinospora*)	[26]
Polycyclic aromatic hydrocarbons	Ring hydroxylases alpha-chain		[19]
	Homotrimeric ring hydroxylases		[25]
RNA	Bet v 1	PR-10	[28,31,32]

The highest diversity of ligand binding activities was shown for members of the Bet v 1 family. The first biochemical activity proposed for PR-10 proteins was as ribonuclease. RNase activity was first shown for PR-10 proteins from ginseng [28] and later demonstrated for a number of PR-10 family members including Bet v 1 [29-32]. A possible nucleic acid binding function was supported by the fact that the most conserved part in the sequences of PR-10 proteins is a glycine-rich loop between strands 2 and 3 [12], a so-called P-loop that is frequently found in nucleotide-binding proteins [33]. However, the conformation of the glycine-rich loop differs from the canonical P-loop structure and the biological significance of the in vitro RNase activity remains controversial [34].

The determination of the crystal structures of PR-10 proteins and their similarities with the START domain prompted researchers to search for ligands fitting into the hydrophobic cavity. A binding activity to the plant steroid hormones brassinosteroids was experimentally determined for Bet v 1 and Pru av 1 [13,35]. A different group of plant hormones, the cytokinins, was shown to bind to Bet v 1 [36,37] as well as to a cytokinin-binding protein from mung bean distantly related to PR-10 [11] and a Bet v 1 homologue from the moss *Physcomitrella patens *[38]. Ligand binding activities of Bet v 1 were extensively examined. In addition to brassinosteroids and cytokinins, the protein was shown to bind flavonoids and fatty acids [36,37]. The Bet v 1 family also contains two groups of enzymes. Hyp-1, a PR-10 from St. John's wort (*Hypericum perforatum*), catalyses the condensation of two molecules of emodin to the bioactive naphthodianthrone hypericin [39]. (S)-Norcoclaurine synthases catalyse the condensation of dopamine and 4-hydroxyphenylacetaldehyde to (S)-norcoclaurine, the first committed step in the biosynthesis of benzylisoquinoline alkaloids such as morphine [9,10].

The types of ligands most frequently determined to bind other Bet v 1-like superfamily members were membrane lipids (Table 4). Binding of cholesterol, phospholipids and ceramide was shown for members of the START and PITP families. A group of uncharacterised eukaryotic proteins from the polyketide cyclase family were annotated in the Uniprot database as binding to coenzyme Q. A function related to membrane lipid binding is binding to membranes, which is part of the biological activity of START and PITP family members, but was also shown for Bet v 1 [40]. CoxG from *Oligotropha carboxidovorans *anchors the CO dehydrogenase to the cytoplasmic membrane [27].

Many bacterial Bet v 1-related proteins exert highly specialised functions such as biosynthesis of secondary metabolites, degradation of aromatic compounds, and antibiotic resistance (Table 4). The structures of several Bet v 1-related prokaryotic proteins were determined in the course of structural genomics project. Hence the biologic function of these proteins remains to be elucidated.

### Collection of a set of non-redundant Bet v 1-related sequences

In order to gain a deeper insight into evolutionary relationships within the Bet v 1 family, we performed a phylogenetic analysis of protein sequences with significant similarity to Bet v 1. The Pfam database (version 22.0, July 2007) listed 383 non-fragment sequences as members of the Pathogenesis-related protein Bet v I family [Pfam:PF00407]. BLAST database searches with representative members of already known subfamilies yielded 42 additional entries not listed in Pfam adding up to 425 different Bet v 1-related sequences. This data set contained a large number of groups of highly similar isoforms and variants from the same species. Thus, the redundancy of the data set was reduced by deleting all sequences with more than 90% identity to any other sequence leaving 221 entries.

Among those, a PR-10-like protein from the moss *Physcomitrella patens *[Uniprot:Q9AXI3] and two genomic sequences from *Arabidopsis thaliana *[Uniprot:Q9SSK9], [Uniprot:Q0WLG8] contained two and one *Arabidopsis *sequence [Uniprot:Q9LQT7] contained four Bet v 1-related domains. These proteins were split into their constituting domains. Seventeen sequences that contained large deletions were removed from the data set. The final dataset contained 210 sequences or domains.

The data set contained a single sequence from outside the plant kingdom, the genomic sequence Mlr1698 from the bacterium *Rhizobium loti *[Uniprot:Q98K03]. In order to identify further bacterial Bet v 1 homologues, a BLAST search with this sequence was performed, which yielded 96 hits before and 58 sequences after reduction of redundancy using a sequence identity threshold of 90%. This data set included three plant sequences. Due to the low sequence identity between bacterial and plant sequences (3–23% identity, 5–43% similarity to Bet v 1; Table 5), both the plant and the bacterial data set were aligned separately.

**Table 5:** Subfamilies of the Bet v 1 family identified by phylogenetic analysis of protein sequences.

Name^a^	Pfam family	Number of sequences	Representative member	Range of percent sequence identity/similarity to Bet v 1
Dicot PR-10	Bet_v_I [Pfam:PF00407]	97	Major pollen allergen Bet v 1; *Betula verrucosa *(white birch) [Uniprot:P15494]	34–100/52–100
Monocot PR-10 type I	Bet_v_I [Pfam:PF00407]	7	PR-10; *Hordeum vulgare *(barley) [Uniprot:Q84QC7]	23–30/37–44
Monocot PR-10 type II	Bet_v_I [Pfam:PF00407]	12	PR-10.1; *Lilium longiflorum *(trumpet lily) [Uniprot:Q9ZPP9]	30–38/50–60
Conifer PR-10	Bet_v_I [Pfam:PF00407]	8	PR-10.1.12; *Pinus monticola *(Western white pine) [Uniprot:Q7X9V8]	32–38/51–57
CSBP	Bet_v_I [Pfam:PF00407]	2	CSBP; *Vigna radiata *(mung bean) [Uniprot:Q9ZWP8]	25–31/45–45
NCS	Bet_v_I [Pfam:PF00407]	8	NCS; *Thalictrum flavum *[Uniprot:Q67A25]	20–25/32–46
MLP/RRP	Bet_v_I [Pfam:PF00407]	60	MLP-15; *Papaver somniferum *(opium poppy) [Uniprot:P19825]	1–32/1–49
Moss PR-10-like	Bet_v_I [Pfam:PF00407]	4	PR-10-like protein; *Physcomitrella patens *(moss) [Uniprot:Q9AXI3]	15–21/32–40
Plant polyketide cyclase-like	Polyketide_cyc [Pfam:PF03364]	10	Expressed protein c17; *Nicotiana tabacum *(common tobacco) [Uniprot:Q53HY7]	10–17/21–32
Bacterial polyketide cyclase/Bet v 1 subfamily	Polyketide_cyc [Pfam:PF03364], Bet_v_I [Pfam:PF00407], DUF680 [Pfam:PF05079]	53	MxaD; *Methylobacterium extorquens *[Uniprot:A7D8I2]	3–23/5–43
Bacterial Bet v 1-related minor group	-	2	Predicted protein Nmul_A1581; *Nitrosospira multiformis* [Uniprot:Q2Y8N9]	14–19/32–37

^a^Abbreviation: CSBP: cytokinin-specific binding proteins; NCS: (S)-norcoclaurine synthases; MLP: major latex proteins; PR-10: pathogenesis-related proteins family 10; RRP: ripening-related proteins.

### Identification of subfamilies in the Bet v 1 family

**Figure 2:**
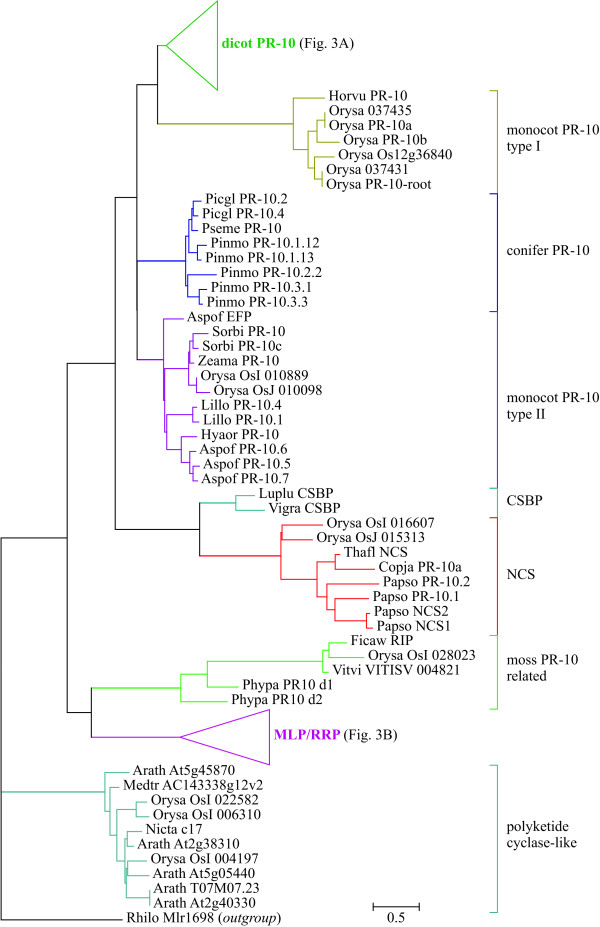
**Distance-based phylogenetic tree of plant protein sequences related to Bet v 1**. The tree was rooted with the only bacterial sequence as outgroup. Uniprot accession numbers of the sequences can be found in Additional file 2.

**Figure 3:**
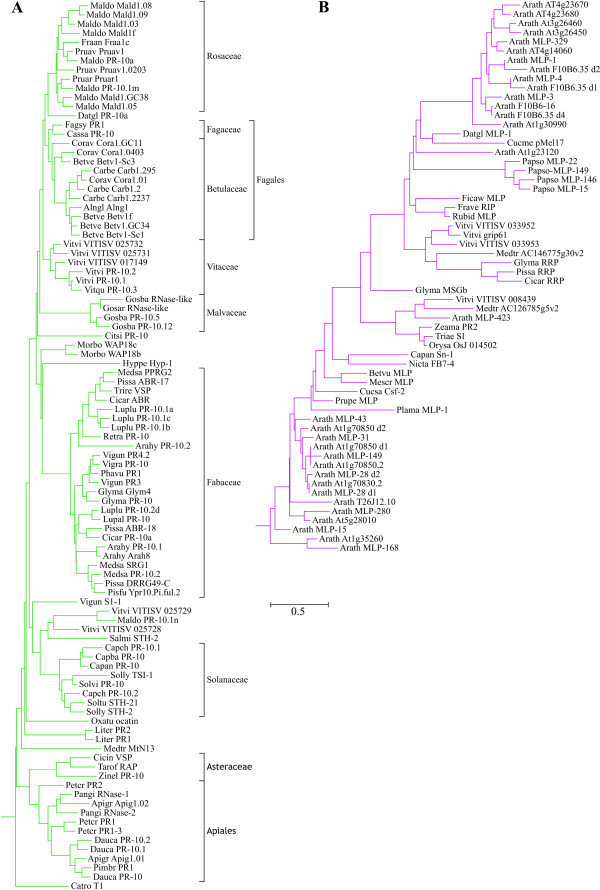
**Distance-based phylogenetic trees of protein sequences from the dicot PR-10 (A) and MLP/RRP (B) subfamilies**. Monophyletic groups comprising proteins from single plant families or orders are labelled in A. Uniprot accession numbers of the sequences can be found in Additional file 2.

**Figure 4:**
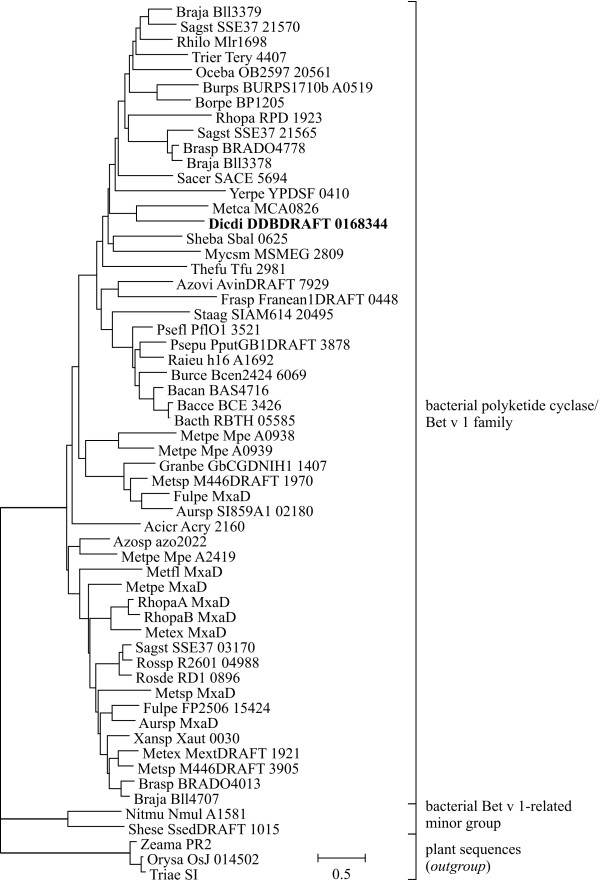
**Distance-based phylogenetic tree of bacterial protein sequences related to Bet v 1**. The tree was rooted with homologous plant sequences as outgroup. Bold: DDBDRAFT_0168344 from *Dictyostelium discoideum*, the only sequence from a species other than plants and bacteria. Uniprot accession numbers of the sequences can be found in Additional file 2.

Two data sets containing 210 and 58 sequences or domains were aligned and distance-based phylogenetic trees were constructed (Figs. 2, 3, 4). No bootstrap analysis could be performed due to the fact that about one third of the bootstrapped data sets resulted in infinite distances for some sequence pairs while the original alignment did not. However, the branches that connected different subfamilies in the tree were generally much longer than the branches within each subfamily, thus rendering the identification of subfamilies straightforward (Figs. 2 and 4).

Eleven subfamilies of the Bet v 1 family were identified (Table 5, Additional file 2). Plant sequences were classified into nine, bacterial sequences into two subfamilies. The only sequence outside these taxa was the genomic sequence DDBDRAFT_0168344 from the slime mould *Dictyostelium discoideum *[Uniprot:Q550X0], which clustered with bacterial sequences (bold in Fig. 4). Members of all plant subfamilies but the polyketide cyclase-like subfamily matched the Bet v 1 family signature [Pfam:PF00407]. The bacterial polyketide cyclase/Bet v 1 subfamily showed no clear match with any Pfam family: some members were related to the Bet v 1 family, others could be aligned with the polyketide cyclase family signature [Pfam:PF03367] or the signature of a domain of unknown function [Pfam:PF05079]. However, the alignment scores were in most cases below the Pfam gathering thresholds.

#### Dicot PR-10 subfamily

The largest subfamily was the dicot PR-10 subfamily with 97 members in the non-redundant data set (Fig. 3A). This group contained two types of proteins: genuine PR-proteins whose expression is upregulated upon pathogen infection, wounding or by abiotic stress, and PR-like proteins whose expression is developmentally regulated (reviewed in [5]). Many proteins from the latter group are expressed in high amounts in pollen, seeds, fruits, or vegetative storage organs. A striking example for this type of PR-like proteins is ocatin [Uniprot:Q8W2B4], the major storage protein of the tubers of the South American crop oca (*Oxalis tuberosa*), which accounts for 40–60% of the total soluble protein content of the tubers [41]. PR-10 proteins are members of small multigene families. However, most PR-10 isoforms cluster exclusively with homologues from the same plant family (Fig. 3A), which can be explained either by proposing multiple independent duplication events in the common ancestors of these plant families or, more probably, by the assumption of a strong concerted evolution of PR-10 loci within each species. A similar conclusion was drawn in a previous publication based on phylogenetic analysis of the much smaller number of PR-10 sequences known at that time [42].

#### PR-10 from monocots and conifers

PR-10 proteins from monocots and conifers are closely related to dicot PR-10 proteins, both with respect to sequence similarity and function. Sequences from monocots were divided into two protein subfamilies, one restricted to cereals and one that contained members from Poaceae and from other plant families such as Asparagaceae and Liliaceae (Fig. 2).

#### Subfamilies related to PR-10

Two small subfamilies were more distantly related to PR-10 (Table 5): the cytokinin-specific binding proteins (CSBP) and the (S)-norcoclaurine synthases (NCS). Members of the CSBP subfamily, which is restricted to legumes, bind the plant hormone cytokinin [43], an activity that has also been shown for some members of the PR-10 subfamily [36]. Proteins with NCS activity were found in *Thalictrum flavum *[10] and in opium poppy (*Papaver somniferum*) [9]. Two additional poppy proteins [Uniprot:Q4QTI9], [Uniprot:Q4QTJ0] as well as a protein from *Coptis japonica *[Uniprot:A2A1A1], a plant closely related to *T. flavum*, have been designated PR-10 but showed close similarities with NCS.

#### MLP/RRP subfamily

The second largest subfamily among plant proteins is the major latex protein/ripening-related protein (MLP/RRP) subfamily with 60 members, 31 of them from *Arabidopsis thaliana *(Fig. 3B). The other 29 sequences distribute into only 21 species from dicots (26 sequences from 18 species) and monocots (3 sequences from 3 species). Members of this subfamily were first described as proteins abundantly expressed in the latex of opium poppy (*Papaver somniferum*) [44]. Most members of the MLP/RRP subfamily are expressed in fruits with their expression upregulated during ripening. Examples are a ripening-induced protein from wild strawberry [45], Csf-2 from cucumber [46] and Sn-1 from bell pepper, whose expression can also be induced by wounding in unripe fruits [47]. A wound-induced transcript from *Mesembryanthemum crystallinum *[Uniprot:O65178] also belongs to this subfamily. The expression of the tobacco cDNA FB7-4 is induced during flower formation [48]. The biological function of the MLP/RRP proteins is still unknown, but the expression patterns suggest a role in defence or stress response.

#### Moss PR-10

The only Bet v 1-related protein found in plants other than Spermatophyta, a protein from the moss *Physcomitrella patents *containing two Bet v 1 domains [38] shows highest similarity to the MLP/RRP subfamily (Fig. 2). Three divergent angiosperm MLP-like sequences cluster with the *P. patens *domains, but contain only a single domain.

#### Polyketide cyclase-like subfamily

A group of plant proteins distantly related to PR-10 as well as MLPs (10–17% identity, 21–32% similarity to Bet v 1; 12–20% identity, 23–33% similarity with poppy MLP-15) matched the Pfam Polyketide cyclase family signature [Pfam:PF03364]. This subfamily contains uncharacterized genomic sequences from tobacco, *Arabidopsis thaliana *and *Medicago trunculata *(Fig. 2).

#### Bacterial Bet v 1-like subfamilies

Bacterial Bet v 1-related sequences were grouped into two subfamilies (Fig. 4), the major one distantly related to the Pfam Bet v 1 as well as Polyketide cyclase families, the minor group, which comprised only two sequences, not matching any Pfam family. Most of the bacterial sequences were derived from uncharacterized genomic clones. One exception is the periplasmic 17 kDa protein MxaD from the methylotrophic bacterium *Methylobacterium extorquens*. This protein enhances the rate of methanol oxidation by stimulating the interaction between methanol oxidase and its electron acceptor, cytochrome c_L _[49].

The distant sequence similarity between plant and bacterial members of the ubiquitous polyketide cyclase family and Bet v 1 family members suggests that the Bet v 1 family evolved from the polyketide cyclases in plants as a response to specialized needs of higher plants with respect to development, stress response, defence, and secondary metabolism. No clear connection between sequence and function was detected with the exception of the NCS subfamily whose members show a common function. In contrast, hormone binding activity was found for members of the PR-10 and CSBP families as well as for a MLP-like moss protein. Similarly, developmentally regulated, pathogen-induced and tissue-specific constitutive expression patterns are equally distributed among members of the PR-10 and MPL/RRP subfamilies with no correlation between phylogenetic relationship and function. Thus, the elucidation of the evolution of the Bet v 1 family will require the identification and functional characterisation of additional Bet v 1-related proteins from plants outside the Spermatophyta.

## Conclusion

**Figure 5:**
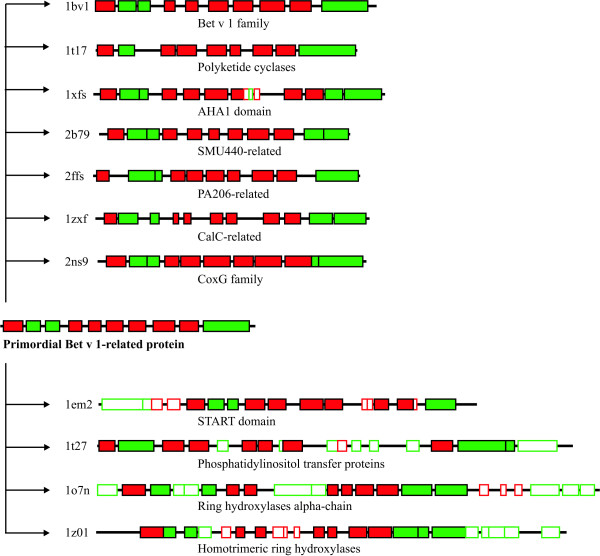
**Secondary structure arrangements of Bet v 1-like superfamily members**. Red: β-strands; green: α-helices; filled rectangles: structural elements homologous to corresponding ones in Bet v 1; open rectangles: insertions compared to Bet v 1. Proteins are identified by their PDB accession numbers. Positions of the secondary structure elements are given as provided by the authors in the PDB files.

A comparison of the structures, functions and taxonomic distributions of members of the Bet v 1-like superfamily leads to the suggestion of the following evolutionary scenario (Fig. 5). A protein possessing the Bet v 1 fold most likely already existed in the last universal common ancestor. The biological function of this protein was probably related to lipid binding, such as trafficking of membrane components. This primordial gene subsequently diverged into the multitude of Bet v 1-related protein families present today, some of which retained the original fold, while others gained novel function by insertion of additional structural elements (Fig. 5, bottom half). The ubiquitous distribution of the polyketide cyclase family and the fact that the structures of its members comprise the minimal Bet v 1 fold without insertion of additional structural elements (Fig. 5) suggest that these proteins are most closely related to the primordial Bet v 1-like protein. Functional diversity within the Bet v 1-like superfamily was also accomplished by fusion to other domains such as DNA binding modules of transcription regulators found as members of several Bet v 1-related families.

During evolution, sequence similarity between members of different families decreased to values that make the prediction of homology unreliable. However, it is unlikely that this fold with the distinctive topology of the β-sheet has evolved more than once. Nevertheless, an architecture characterised by an anti-parallel β-sheet wrapped around a long α-helix forming a large cavity was invented at least twice during evolution of lipid binding proteins. Our structural similarity search showed that a family of oxysterol binding proteins adopts a fold similar to Bet v 1 at first sight, but with a different topology.

An intriguing aspect of the evolution of the Bet v 1-like superfamily is the evolution of allergenicity. Bet v 1 from birch pollen and possibly its close homologues from other Fagales tree pollen are the only proteins within this ubiquitously distributed superfamily known to be capable of initiating an allergic immune response in humans [50]. This rare occurrence of allergenicity is in sharp contrast to the distribution of allergens within the most important superfamily of allergens, the prolamins, in which numerous families of allergens were identified, such as the 2S albumins, prolamin storage proteins, non-specific lipid transfer proteins, bifunctional inhibitors, indolines, and α-globulins [50,51]. Among the still unknown factors that render a protein allergenic, structural features are frequently discussed. Hence, the comparison of Bet v 1 with its non-allergenic structural homologues offers the possibility to shed light on such features, thus paving the way for a deeper understanding of the allergic sensitisation process and the development of novel concepts for prevention and treatment of allergy.

## Methods

### Search for structural homologues of Bet v 1

The structural superfamily of which Bet v 1 is a member has been defined in the SCOP database version 1.71 [52] as the Bet v 1-like superfamily [SCOP:d.129.3]. Its member families were used as a starting point for the search for Bet v 1-related structures. An all against all structural alignment within each family yielded a representative structure for each family. Subsequently, each representative structure was compared to the complete PDB database. All structural comparisons were performed with psc++, an improved version of the ProSup structural alignment programme [53]. During the structural alignment, the programme maximises the number of aligned backbone atoms while confining the root mean square distance (RMSD) of the aligned residues to values below 3.0 Å. The results of the PDB searches were sorted by the number of aligned residues.

All structures with more than half of their residues aligned with one of the query structures were manually classified into structural families using a combination of several criteria. (1) A Bet v 1-like topology of the structure was confirmed by visual inspection. (2) The structural similarities to the query structures were compared with respect to the number of aligned residues. (3) Sequence similarity to members of known protein families was checked using a BLAST search against the PDB with an expect value threshold of 10^-3^. If the BLAST search yielded no significant result, the sequence was compared with the Pfam database (version 22.0, July 2007) using the sequence search tool provided on the Pfam website [54]. Alignment scores above the Pfam gathering thresholds were counted significant.

### Sequence comparison among Bet v 1-like superfamily members

Sequence identities and similarities of Bet v 1-like superfamily members to Bet v 1 were determined using the global sequence alignment programme Needle from the EMBOSS package provided by the Center of Integrative Bioinformatics, University of Vienna, Austria. After matching structural families with sequence-based families of the Pfam database (version 22.0, July 2007, [55]), partial sequences matching the Pfam hidden Markov models (HMMs) were downloaded from the Pfam website and aligned with partial sequence 1–155 of Bet v 1 [Uniprot:P15494]. For an all-against-all comparison of sequences from different Bet v 1-related families, HMM-matching partial sequences of representative members of each family (those sequences that were used to construct the Pfam family seed alignment) were downloaded from the Pfam website. For each pair of families the median sequence identities and similarities were calculated.

### Compilation of Bet v 1-related sequences

The starting point for the collection of Bet v 1-related sequences were the non-fragment Uniprot entries that were classified as members of the Pathogenesis-related protein Bet v I family [Pfam:PF00407] in the Pfam database (version 22.0, July 2007, [55]). A provisional alignment and a neighbour-joining tree were generated using ClustalX 1.83 [56] and visualised using MEGA 4 [57]. Representative sequences from different subfamilies identified in this tree were used as queries for BLASTP similarity searches within non-fragment sequences from the Uniprot database [58] in order to find additional sequences not included in Pfam. An expect value threshold of 10^-3 ^was used.

Redundancy within the resulting sequence set was reduced by removing all sequences with identity above 90% to any other sequence using the remove redundancy tool located at the Swiss Institute of Bioinformatics [59]. Long sequences were compared to the Pfam database and split into single domains if they contained multiple Bet v 1-related domains. Short sequences that matched only one of the Bet v 1 fragment HMMs provided by the Pfam database were removed from the list. A second provisional alignment of the remaining sequences was manually inspected and sequences that contained large (> 10 residues) deletions compared to the majority of closely-related sequences were removed.

Bacterial homologues of Bet v 1 were identified by a BLASTP search with the genomic sequence Mlr1698 from *Rhizobium loti *[Uniprot:Q98K03], the only bacterial sequence found by BLASTP searches with plant sequences. Redundancy reduction was performed as described above.

### Multiple sequence alignment and phylogenetic tree building

Plant and bacterial sequences were aligned separately using ClustalX with default parameters. The resulting alignments were truncated on the N and C-terminal ends up to the first columns in which the majority of the sequences contained a residue. Pairwise distance matrices applying the Jones-Taylor-Thornton model of amino acid replacement were calculated using the programme Protdist from the PHYLIP 3.67 package [60]. Distance-based trees using the method of Fitch and Margoliash were calculated using the PHYLIP programme Fitch with the global search option activated. The trees were visualised using MEGA 4 [57].

Sequence identities and similarities of Bet v 1 family members to Bet v 1 [Uniprot:P15494] were determined using the global sequence alignment programme Needle from the EMBOSS package provided by the Center of Integrative Bioinformatics, University of Vienna, Austria.

## Abbreviations

AHA1: activator of Hsp90 ATPase homolog 1; CSBP: cytokinin-specific binding protein; HMM: hidden Markov model; MLN64: metastatic lymph node protein 64; MLP: major latex protein; NCS: (S)-norcoclaurine synthase; PDB: Protein Data Bank; PITP: phosphatidylinositol transfer protein; PR: pathogenesis-related; RMSD: root mean square distance; RRP: ripening-related protein; SCOP: Structural Classification of Proteins; STAR: steroidogenic acute regulatory protein; START: STAR lipid transfer domain.

## Authors' contributions

CR performed the phylogenetic analysis of Bet v 1-related sequences, critically reviewed the results of the structural comparison, and wrote the manuscript. PL designed and performed the search for Bet v 1-related structures and participated in writing the manuscript. HB conceived the study, participated in its design and in writing the manuscript. All authors critically read and approved the final manuscript.

## Supplementary Material

Additional file 1 PDB entries of Bet v 1-related structures. Click here for file

Additional file 2 Names, Uniprot accession numbers and sources of proteins in Figs. 2 through 4. Click here for file
